# PURPOSe study: understanding the burden of stillbirths in south Asia

**DOI:** 10.1016/S2214-109X(22)00218-2

**Published:** 2022-06-14

**Authors:** Joao Paulo Souza, Rajiv Bahl

**Affiliations:** a Department of Social Medicine, Ribeirao Preto Medical School, University of Sao Paulo, Ribeirao Preto, Brazil; b Department of Maternal, Newborn, Child and Adolescent Health and Ageing, World Health Organization, Geneva, CH-1211, Switzerland

Stillbirth prevention is a global health priority and a crucial step towards better maternal and newborn health and wellbeing.^1^ In 2019, 2 million babies were stillborn, with over three-quarters of these stillbirths occurring in sub-Saharan Africa and south Asia.^2^ However, progress has been slow, and unless there is a substantial acceleration in progress, the Sustainable Development Goal target 3.2 and Every Newborn Action Plan target of 12 stillbirths per 1000 births will not be met by 2030.^3^ Slow progress is partly due to the limited emphasis on stillbirth reduction in maternal and child health programmes and a paucity of accurate, complete, and actionable information on stillbirths, particularly in high-burden areas.^1^, ^4^

In *The Lancet Global Health*, Elizabeth M McClure and colleagues^5^ present the findings of a large prospective observational study on the causes of stillbirth in India and Pakistan (the PURPOSe study). In this study, prospective surveillance was used to identify all women with fetal deaths or stillbirths attending the participating facilities during the study period. The causes of death of stillborn babies weighing more than 1000 g were identified using multiple sources of information, including interviews with mothers, hospital records review, physical examination, laboratory tests, placental pathology, minimally invasive tissue samples, and other microbiological methods. Using standard operating procedures, a panel of experts ascertained the primary maternal, fetal, and placental biomedical causes of death for 611 stillborn babies. The authors found that intrauterine hypoxia (72%) and congenital infections (13%) were the two predominant causes of stillbirth. This ascertainment was largely based on evidence of placental malperfusion (47%), abruption (15%), or infection (14%). The most important maternal causes were hypertensive disorder (36%), anaemia (11%), and infection (5%); a third of stillbirths did not have any associated maternal condition. A key limitation of the study was the exclusion of stillbirths weighing <1000 g, which implies that the causes among very preterm and severely growth-restricted babies were under-represented.

Compared with previous studies,^6^, ^7^ PURPOSe adds precision to determining the causes of fetal death. The large sample size of this study, coupled with the prospective surveillance and the high rate of success in doing placental pathology, contributes to making PURPOSe a crucial source of evidence on the biomedical causes of fetal mortality, particularly in south Asia. As with maternal and newborn mortality, biomedical causes often represent the embodiment of the complex interplay of ecosocial forces with individual factors.^8^, ^9^ As a product of this interplay, stillbirths result from a long multifactorial process with missed opportunities to interrupt the pathway to death. In this pathway, the health system has the unique role of delivering quality and effective interventions to promote health and prevent and treat complications. PURPOSe and the body of evidence surrounding stillbirths point to the importance of primary prevention, early detection, and treatment of hypertensive disorders of pregnancy, maternal anaemia, and infections. Quality intrapartum care is crucial to reducing intrapartum stillbirths, which still contribute to at least a third of the burden of stillbirths in low-income and middle-income countries. Respectful antepartum and intrapartum care programmes are essential platforms for delivering effective health interventions.^10^, ^11^ Health system interventions to provide continuity of care across programmes and services (eg, midwife-led care models to ensure continuity of care between antenatal care and intrapartum care) should not be overlooked.^12^

The contribution of PURPOSe to understanding the causes of stillbirths in south Asia calls for a similar large study in sub-Saharan Africa, including stillbirths weighing less than 1000 g. Research to develop improved methods for early identification of placental malperfusion and placental infection is needed. Most importantly, a series of intervention trials addressing the major causes maternal, placental, and fetal causes of stillbirth in south Asia and sub-Saharan Africa are urgently needed.

The global burden of stillbirths is a silent disaster that has long-term consequences for women, families, and their communities. Many stillbirths are preventable, and the interventions needed to prevent stillbirths are integrated into efforts to improve the quality of care during pregnancy, childbirth, and the postnatal period. The health-care community need intentional leadership and a greater commitment to efforts to implement and scale-up existing and new solutions to reduce stillbirths within health systems in low-resource settings. Purposefully aiming to reduce stillbirths will ultimately improve maternal and newborn health and wellbeing. By tackling stillbirths, the health-care community will achieve an acceleration toward the Sustainable Development Goals.

We declare no competing interests.

## References

[1] de Bernis L, Kinney MV, Stones W (2016). Stillbirths: ending preventable deaths by 2030. Lancet.

[2] Hug L, You D, Blencowe H (2021). Global, regional, and national estimates and trends in stillbirths from 2000 to 2019: a systematic assessment. Lancet.

[3] WHO (2017).

[4] Kiguli J, Kirunda R, Nabaliisa J, Nalwadda CK (2021). Inadequate evidence on stillbirths: rethinking public health. Lancet.

[5] McClure EM, Saleem S, Goudar SS (2022). The causes of stillbirths in south Asia: results from a prospective study in India and Pakistan (PURPOSe). Lancet Glob Health.

[6] Lawn JE, Blencowe H, Waiswa P (2016). Stillbirths: rates, risk factors, and acceleration towards 2030. Lancet.

[7] Ahmed I, Ali SM, Amenga-Etego S (2018). Population-based rates, timing, and causes of maternal deaths, stillbirths, and neonatal deaths in south Asia and sub-Saharan Africa: a multi-country prospective cohort study. Lancet Glob Health.

[8] Krieger N (2005). Embodiment: a conceptual glossary for epidemiology. J Epidemiol Community Health.

[9] Souza JP, Bellissimo-Rodrigues F, Santos LLD (2020). Maternal mortality: an eco-social phenomenon that calls for systemic action. Rev Bras Ginecol Obstet.

[10] (2016).

[11] WHO (2018).

[12] Miller S, Abalos E, Chamillard M (2016). Beyond too little, too late and too much, too soon: a pathway towards evidence-based, respectful maternity care worldwide. Lancet.

